# Suppression of miR-26a attenuates physiological disturbances arising from exposure of Nile tilapia (*Oreochromis niloticus*) to ammonia

**DOI:** 10.1242/bio.029082

**Published:** 2018-04-03

**Authors:** Yan Zhao, Haotian Zhou, Christian Larbi Ayisi, Yan Wang, Jun Wang, Xiaowu Chen, Jinling Zhao

**Affiliations:** 1 Key Laboratory of Freshwater Aquatic Genetic Resources, Ministry of Agriculture, Shanghai Ocean University, 201306, Shanghai, China; 2 National Demonstration Center for Experimental Fisheries Science Education, Shanghai Ocean University, 201306, Shanghai, China; 3 Shanghai Collaborative Innovation for Aquatic Animal Genetics and Breeding, Shanghai Ocean University, 201306, Shanghai, China

**Keywords:** MiRNA, Ammonia stress, Nile tilapia, Physiological performance

## Abstract

MicroRNAs may affect stress responses because they act as rapid responders at the post-translation level. In this study, we found that miR-26a is abundantly expressed in the brain and gill tissues of tilapia. Expression of miR-26a in the brain decreased significantly with increasing ammonia concentrations using stem-loop qPCR. To analyze the function of miRNA *in vivo*, miR-26a was stably knocked down with an antagomir in tilapia. Following ammonia challenge, miR-26a antagomir treatment significantly suppressed blood ammonia/[Cl^−^]/[K^+^] concentration and the reactive oxygen species production, while it markedly enhanced glutamine accumulation and antioxidant enzyme activity in the brain of tilapia, indicating that miR-26a may be involved in the remission of physiological disturbances resulting from ammonia stress. We strongly conclude that there is a direct link between miR-26a and the responses to ammonia in tilapia. Furthermore, bioinformatics analysis and luciferase assays demonstrated that miR-26a regulates *HSP70* (heat shock protein 70) and *GS* (glutamine synthetase) expression by targeting their 3′-UTR and that the suppression of miR-26a could increase the intracellular level of *HSP70* and *GS in vivo*.

## Abstract (precis)

**Summary:** Our work increases the available information about the regulation of miR-26a and indicates that miR-26a may be involved in the remission of physiological disorders upon ammonia stress in tilapia.

## INTRODUCTION

Nile tilapia (*Oreochromis niloticus*) is a widely popular fish that accounts for a high proportion of the world's aquaculture yields due to its excellent growth rates and prominent adaptability to various environmental conditions (Raulw et al., 2010). Among all of the water-quality parameters that affect and influence fish behavior and health, ammonia is one of the most common and important. Therefore, an extensive understanding of the response to ammonia stress and ammonia tolerance in tilapia may provide both physiologists and fish breeding experts with valuable information to improve aquaculture.

MicroRNAs are a subclass of small non-coding RNAs that are produced by Dicer from precursors with a characteristic hairpin structure. They are complementary to one or more mRNAs with the binding of specific sequences in the 3′ untranslated region (UTR) of the target genes, and they down-regulate gene expression primarily through translational repression, mRNA cleavage, or deadenylation. MicroRNAs are involved in regulating various cellular processes, including cell fate determination, apoptosis, and tumor suppression (Bartel, 2004). Furthermore, tissue-specific microRNAs that are specifically expressed in particular tissues may have particular functions (Guo et al., 2014; Kim et al., 2006; Makeyev and Maniatis, 2008).

The brain is one of the most important organs involved in ammonia tolerance. It can avoid ammonia toxicity by detoxifying ammonia to products such as glutamine in fish (Chew et al., 2001; Ip et al., 2001a,b). The miR-26 family shows highly restricted expression in the brain of many animals (Smirnova et al., 2005). Moreover, miR-26a appears to be a vertebrate-specific microRNA (https://en.wikipedia.org/wiki/Mir-26_microRNA_precursor_family) that is required for synaptic-plasticity maintenance and spine enlargement (Gu et al., 2015). The role of miR-26a in response to ammonia stress in fish has not yet been clarified, whereas recent studies had clearly shown that 43 miRNAs including miR-26 were down-regulated by ammonia exposure in the brain of rat (Oenarto et al., 2016). We therefore speculate that miR-26a may be implicated in mediating the ammonia response in Nile tilapia.

Our results support the conclusion that miR-26a is abundantly expressed brain and gill tissues of tilapia. Ammonia stress leads to a remarkable decrease in miR-26a level. miR-26a knockdown can influence the physiological performance against high ammonia in tilapia. Thus, there is a direct link between miR-26a and the responses to ammonia stress in tilapia. Furthermore, we have identified *HSP70* (heat shock protein 70) and *GS* (glutamine synthetase) as the target genes of miR-26a. miR-26a acts directly at the 3′-UTR of *HSP70* and *GS* respectively, thus suppressing the expression of *HSP70* and *GS* mRNA *in vivo*.

## RESULTS

### Distribution of miR-26a

**Fig. 1.**
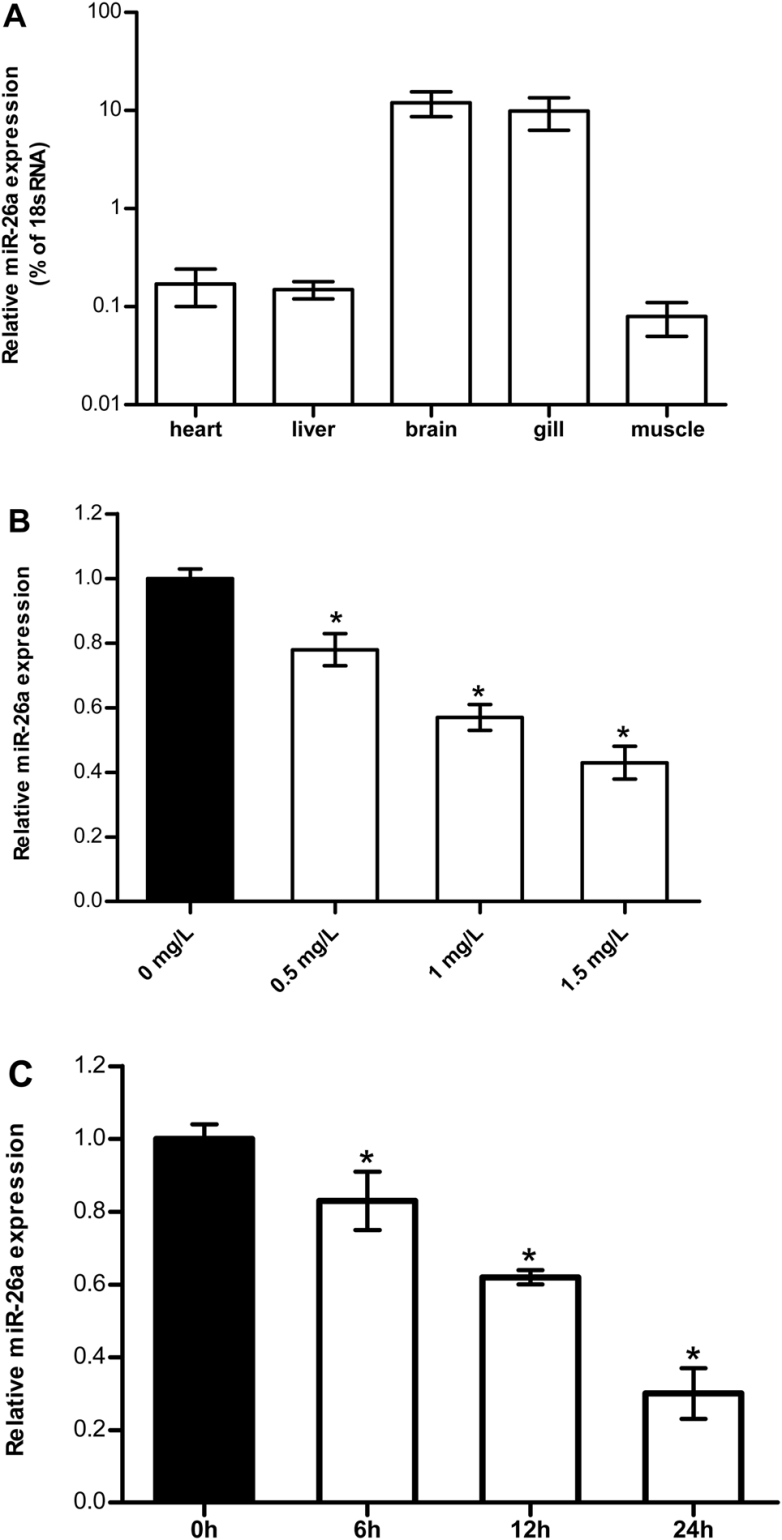
**miR-26a expression pattern.** (A) Tissue (heart, liver, brain, gill and muscle) of fishes that were maintained in freshwater were sampled. The expression of miR-26a was detected using stem-loop PCRs. 18S rRNA was used as the internal control. Each experiment was conducted in triplicate with three sampled fishes. The values are presented as the means±s.d. (*P<0.05, *n*=9). Data were compared using a one-way analysis of variance with the Least-Significant difference post hoc test under homogeneity of variance. (B) Tilapia were exposed to different ammonia concentrations (0, 0.5, 1.0, and 1.5 mg l^−1^) for 6 h. The group exposed to 0 mg/l ammonia concentration was taken as the control group. (C) Tilapia were exposed to 1.0 mg l^−1^ ammonia concentration for 0, 6, 12, and 24 h. The group exposed to 1.0 mg/l for 0 h was taken as the control group. The data (Fig. 1B,C) were expressed as the relative change compared with the control group. The values are presented as the means±s.d. (*P<0.05, *n*=9 fish). Data were compared using a one-way analysis of variance with the Least-Significant difference post hoc test under homogeneity of variance.

The tissue distribution of miR-26a was detected using qRT-PCR. The results suggested that miR-26a was expressed primarily in the brain and gill (Fig. 1A).

### Ammonia exposure and quantification of miR-26a in brain

The expression pattern of miR-26a in fish under different ammonia exposures (0, 0.5, 1.0, and 1.5 mg l^−1^) was determined. The level of miR-26a in the brains showed down-regulation compared with the normal condition by stem-loop qPCR. The relative expression of miR-26a is negatively correlated with the ammonia concentrations. The expression of miR-26a was significantly decreased with increasing ammonia concentrations up to 1.5 mg l^−1^ (Fig. 1B). In addition, ammonia challenge led to a significant reduction in the miR-26a level in a time-dependent manner. The suppression of miR-26a expression was observed as early as 6 h after ammonia stress and as late as 24 h (Fig. 1C).

### miR-26a knockdown influences response to ammonia stress in tilapia

**Fig. 2.**
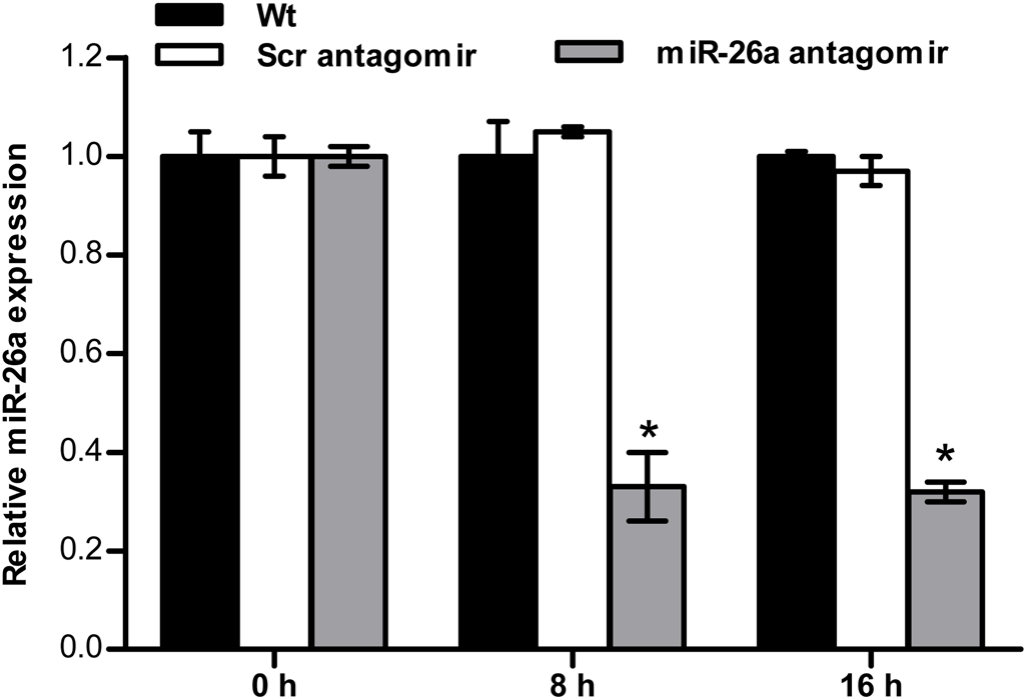
**miR-26a antagomir restrained the endogenous expression of miR-26a.** To check whether the antagomir injection had an effect on the expression of miR-26a, fish were injected with PBS (Control, Wt), nonspecific scrambled antagomir (Scr antagomir) or miR-26a antagomir without ammonia exposure. 18S rRNA was used as the internal control. The relative expression of miR-26a was suppressed 8 h or 16 h after antagomir injection. The data are expressed as the relative change compared with Wt group. The values are presented as the means±s.d. (*P<0.05, *n*=6 fish). Data were compared using a one-way analysis of variance with the Least-Significant difference post hoc test under homogeneity of variance.

To investigate the function of miR-26a in response to ammonia stress *in vivo*, we stably knocked down miR-26a in tilapia with an antagomir specific to this microRNA. The endogenous expression of miR-26a could be restrained by miR-26a antagomir but not scramble antagomir (Fig. 2).

**Fig. 3.**
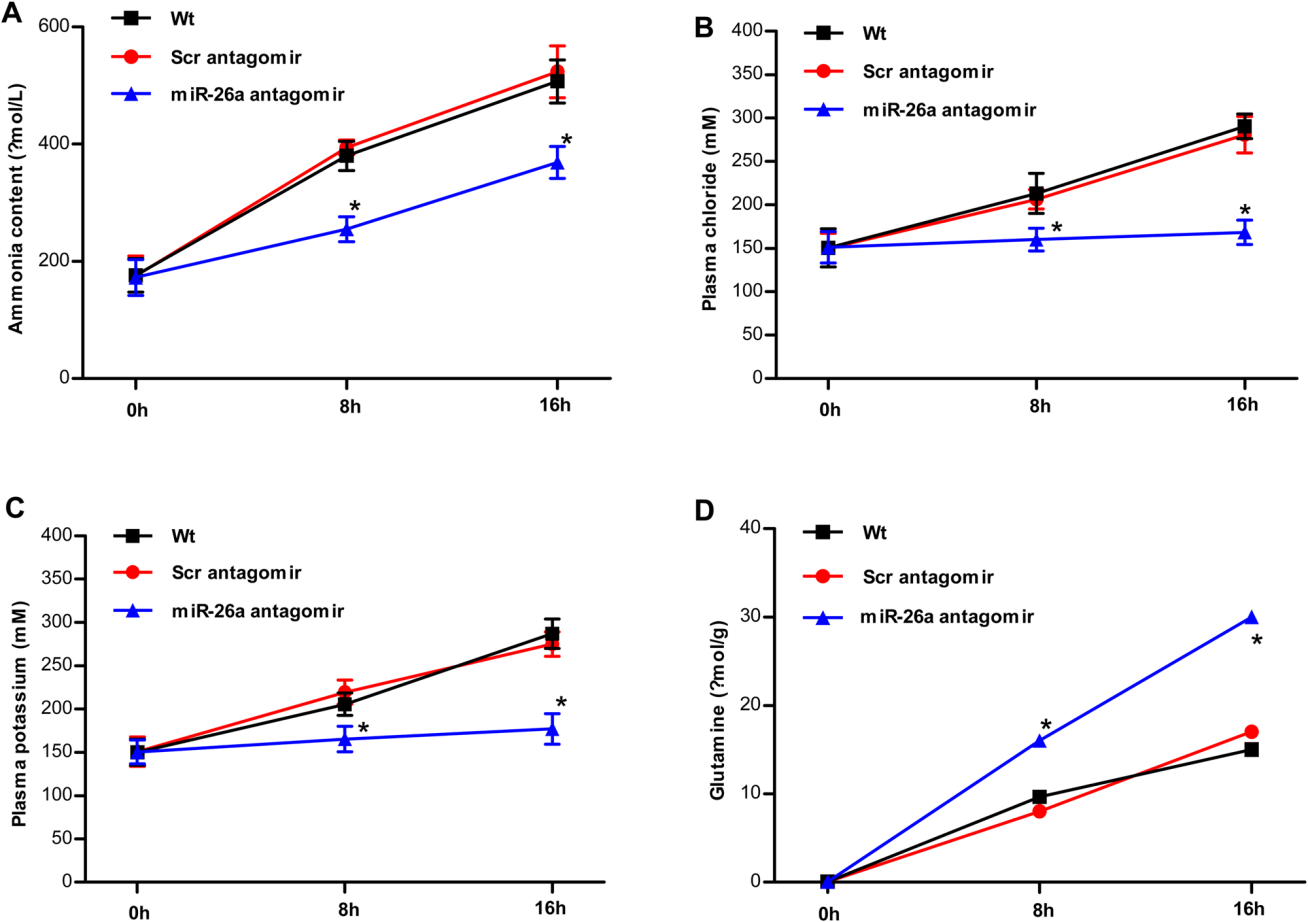
**miR-26a knockdown influences blood ammonia, [Cl−]/[K+] and glutamine concentrations upon ammonia stress in tilapia.** After 48 h, fish that were injected with PBS (Wt), nonspecific scrambled antagomir (Scr antagomir) or miR-26a antagomir were exposed to ammonia. The group injected with PBS served as control. Blood ammonia, [Cl−]/[K+] and brain glutamine concentrations were shown in A-D. Each experiment was conducted in triplicate. The values are presented as the means±s.d. (*P<0.05, *n*=6). Data were compared using a one-way analysis of variance with the Least-Significant difference post hoc test under homogeneity of variance.

There was an increase in ammonia, [Cl^−^]/[K^+^] and glutamine concentrations in all groups during the experimental period when fish were exposed to 1.0 mg l^−1^ ammonia. Compared to the wild type or group administered with scrambled antagomir, concentrations of ammonia, [Cl^−^] and [K^+^] were significantly lower in the group administered with antagomir-26a. On the other hand, glutamine concentration was significantly higher in the group administered with antagomir-26a than those of the wild type or scrambled antagomir group. These data indicate that the miR-26a loss of function influences the plasma ion balance in tilapia (Fig. 3A-D).

**Fig. 4.**
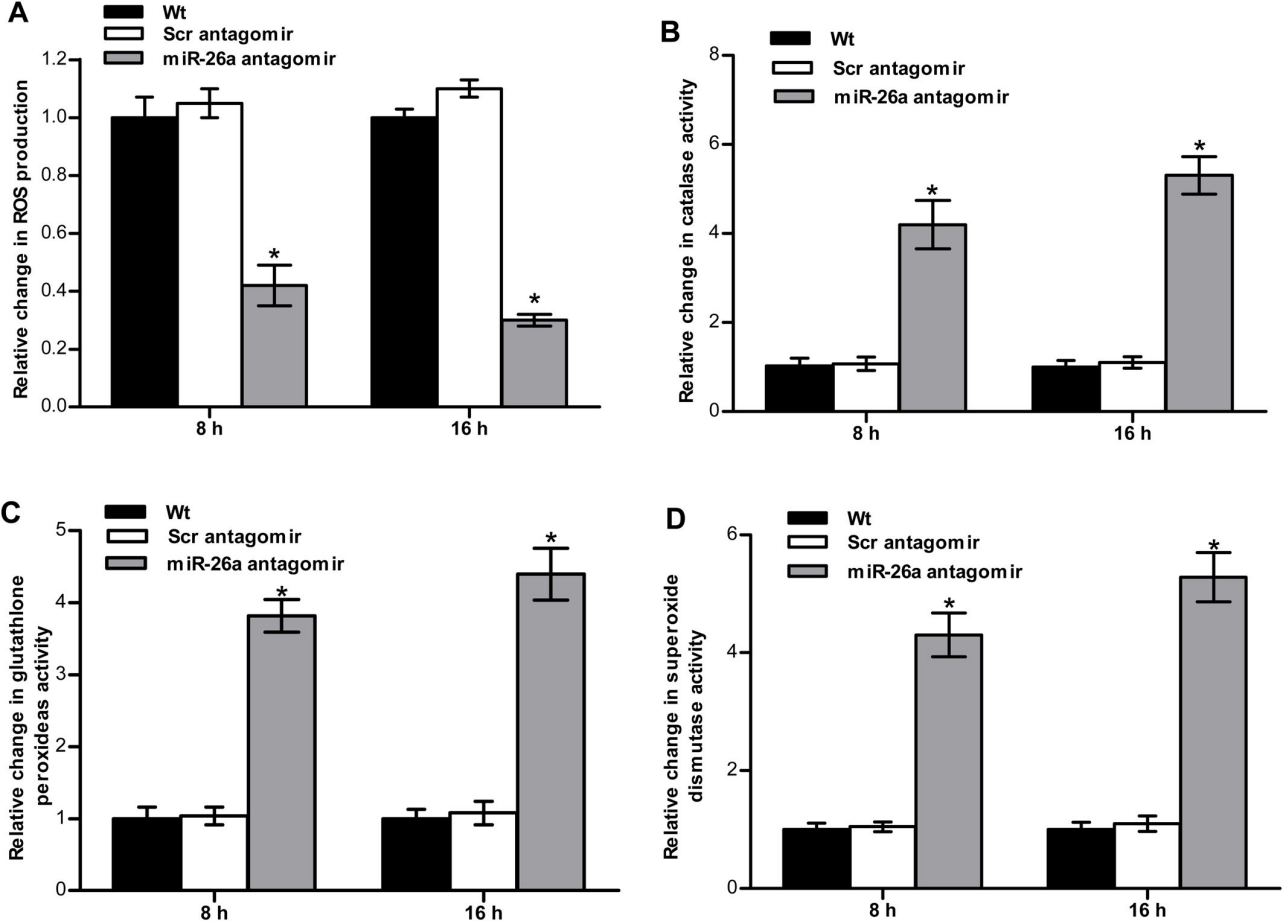
**miR-26a knockdown influences ROS, CAT, GPX and SOD upon ammonia stress in tilapia.** The fish were treated as described in Fig. 3. ROS amount, catalase, glutathione peroxidase, and superoxide dismutase activities are shown in A-D. The values are presented as the means±s.d. (*P<0.05, *n*=6). Data were compared using a one-way analysis of variance with the Least-Significant difference post hoc test under homogeneity of variance.

Ammonia has been reported to induce oxidative stress in the brain (Ching et al., 2009). Thus, we examined whether the miR-26a loss of function influenced the oxidative stress response in the brain of tilapia. The wild-type or scrambled antagomir treatment did not alter the reactive oxygen species (ROS) production, whereas miR-26a antagomir treatment markedly suppressed the ROS production in tilapia (Fig. 4A). Superoxide dismutase (SOD), glutathione peroxidase (GPX) and catalase (CAT) are three major antioxidant enzymes that play crucial roles in the elimination of ROS (Vaziri et al., 2003). miR-26a antagomir treatment significantly enhanced the activities of these antioxidant enzymes (Fig. 4B-D). Hence, these results show that the miR-26a loss of function could strengthen the response to oxidative stress *in vivo*.

### Identification of miR-26a target genes in Tilapia

We attempted to identify miR-26a target genes that are implicated in the response to ammonia stress. Our previous study found that the expression of *HSP70* and *GS* was increased after high ammonia exposure in tilapia. Bioinformatics analysis (http://www.targetscan.org/fish_62/) indicated that there is uninterrupted base pairing between the seed sequence of miR-26a and 3′-UTR of *HSP70*/*GS*. According to this understanding, we considered *HSP70* and *GS* to be potential target genes of miR-26a.

**Fig. 5.**
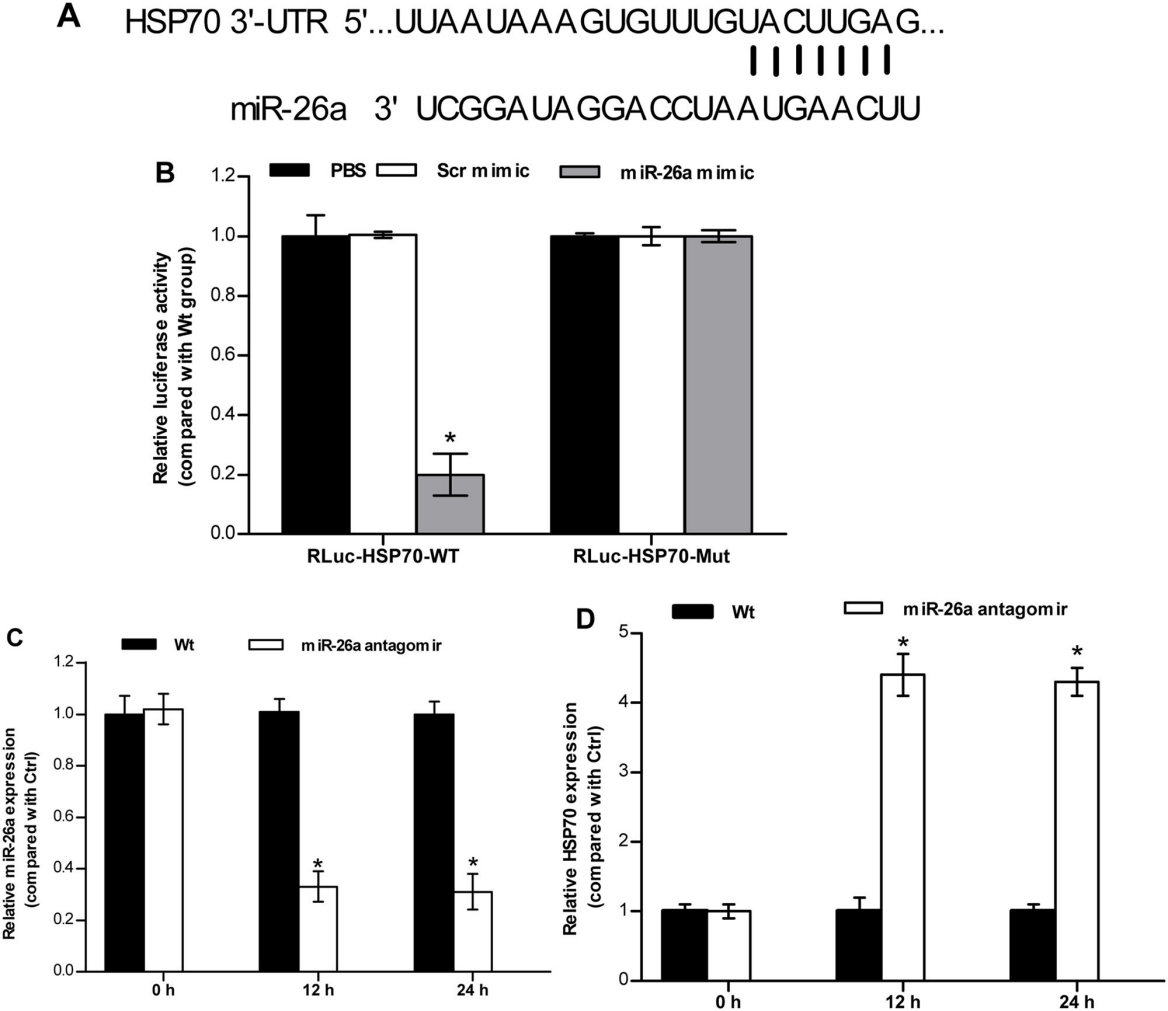
**miR-26a directly targeting HSP70 3′-UTR.** (A) Alignment of the miR-26a sequence and its target in the 3′-UTR region of *HSP70* mRNA. (B) Luciferase assays were carried out to address whether *HSP70* is directly targeted by miR-26a. HEK 293T cells were cotransfected with either the wild-type 3′-UTR of *HSP70* gene (RLuc-HSP70-WT) or mutant UTR of *HSP70* gene (RLuc-HSP70-Mut), together with PBS, scrambled mimic (Scr mimic) and miR-26a mimic. Luciferase activity was assessed 24 h after transfection using the Luciferase Assay Kit. Results are expressed as mean±s.d. of four independent experiments. (C,D) Fish maintained in freshwater were injected with PBS (Wt), nonspecific scrambled antagomir (Scr antagomir) or miR-26a antagomir. The relative expression of miR-26a (C) and *HSP70* (D) was detected using qRT-PCRs. The data are shown as the relative change compared with the control group. The values are presented as the means±s.d. (*P<0.05, *n*=6 fish). Data were compared using a one-way analysis of variance with the Least-Significant difference post hoc test under homogeneity of variance.

**Fig. 6.**
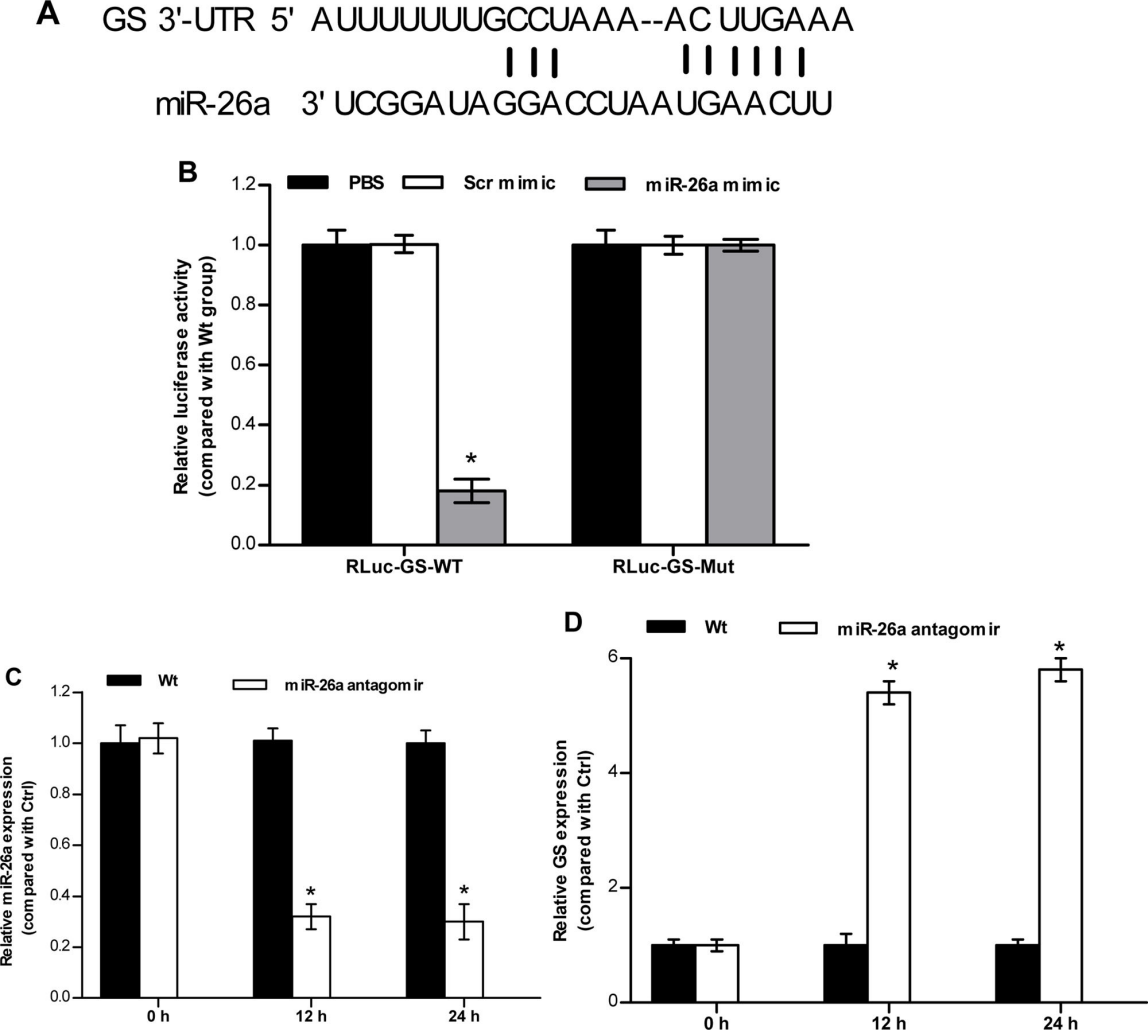
**miR-26a directly targeting GS 3′-UTR.** (A) Alignment of the miR-26a sequence and its target in the 3′-UTR region of *GS* mRNA. (B) Luciferase assays were carried out as mentioned above to address whether *GS* is directly targeted by miR-26a. (C,D) Fish that were maintained in freshwater were injected with PBS (Wt), nonspecific scrambled antagomir (Scr antagomir) or miR-26a antagomir. The relative expression of miR-26a (C) and *GS* (D) was detected using qRT-PCRs. The values are presented as the means±s.d. (*P<0.05, *n*=6 fish). Details of the statistical method were the same as mentioned in Fig. 5.

To validate if miR-26a may truly bind its target site in the 3′-UTR region of *HSP70*/*GS* mRNA (Figs 5A and 6A), we performed luciferase assay experiments and site-directed mutagenesis. HEK 293T cells were co-transfected with the luciferase construct, wild-type 3′-UTR of *HSP70*/*GS* mRNA, mutant 3′-UTR of *HSP70*/*GS* mRNA, and either a miR-26a mimic or a mimic-negative control. In cells overexpressing the miR-26a mimic, the luciferase activity of the wild-type *HSP70*/*GS* reporter was significantly lower than that of the negative control (Figs 5B and 6B). Site-directed mutagenesis of the putative miR-26a-binding region (mutant 3′-UTR of *HSP70*/*GS* mRNA) prevented the inhibitory effect of miR-26a mimic co-transfection (Figs 5B and 6B). These data suggest that miR-26a can specifically bind to the 3′-UTR of *HSP70*/*GS* to suppress protein translation.

We also employed an antagomir strategy to analyze miRNA knockdown *in vivo*. Antagomir administration, but not PBS treatment, successfully reduced endogenous miR-26a expression in the brain (Figs 5C and 6C). Further, *HSP70*/*GS* expression was significantly elevated in the brain (Figs 5D and 6D). Obviously, there was an inverse correlation between miR-26a and *HSP70*/*GS*. These findings indicate that the expression of *HSP70*/*GS* can be directly regulated by miR-26a *in vivo*. In other words, miR-26a targets the 3′-UTR of *HSP70*/*GS* and endogenously inhibits *HSP70*/*GS* expression.

## DISCUSSION

High concentration of ammonia in water is a major environmental stress in fish culture, and it is highly toxic to aquatic vertebrates because it damages the central nervous system (Dosdat et al., 2003; Randall and Tsui, 2002). Identification of genes that could enhance ammonia tolerance of fish is necessary for aquaculture. MicroRNAs are well-known regulatory factors of many biological processes, including the response to biotic and abiotic stresses. Indeed, knowledge about miRNA-mediated stress regulatory networks would provide valuable information to generate genetically modified fish. Recently, many experiments support the view that miRNAs have emerged as important modulators in the ammonia stress response. Forty-three miRNAs that were down-regulated by ammonia exposure in cultured rat astrocytes were detected. Among these miRNAs, miR-31a-5p, miR-221-3p/-5p, miR-222-3p, miR-326-3p and miR-365-3p could mediate the expression of heme oxygenase 1 gene, which often acts as a biomarker for oxidative/nitrosative stress (Oenarto et al., 2016). miR-19b negatively regulated urea synthesis, which is essential for nitrogen homeostasis and ammonia detoxification, by targeting *SIRT5* in weaned piglets (Sun et al., 2016a). miR-21, which was predicted to target several immune-related genes, was significantly down-regulated following ammonia exposure in blunt snout bream (*Megalobrama amblycephala*) (Sun et al., 2016b). In this study, we found an important role of miR-26a in Nile tilapia: decreased expression of endogenous miR-26a could attenuate physiological disturbances after ammonia exposure. This result provides further evidence that miRNAs are important players in the acclimation response of tilapia to high ammonia concentrations.

The underlying mechanism of ammonia tolerance in fish has been studied for decades. The work of Randall, Ip, Wright and colleagues have shed a lot of light on this question (Randall and Tusi (2002); Randall et al., 1999, 2004; Ip et al., 2001a,b, 2004a,b; Sinha et al., 2013). When the blood ammonia concentrations increase in the presence of high environmental ammonia levels, the intracellular ammonia concentrations in the brain also increase. Thus, ammonia could cross the blood–brain barrier in teleost fish (Chew et al., 2001). Ammonia may stimulate ventilation, which can be sensed by the brain, and the concentrations of brain ammonia are mediated by the metabolic network in rainbow trout (Zhang, et al., 2013). In response to acute, high environmental ammonia exposure, tilapia undergoes a crisis period in which there is an increase in ammonia concentration and ion exchange. Tilapia could tolerate or avoid ammonia toxicity by transforming ammonia to less toxic ingredients, including glutamine in the brain (Randall and Tsui, 2002). In our study, miR-26a is highly expressed in the brain. The miR-26a knockdown group exhibited a lower ammonia/[Cl^−^]/[K^+^] concentration and higher glutamine concentration than the control group, indicating that the miR-26a knockdown group would undergo less serious ionic imbalance during the crisis stage and miR-26a silencing could enhance ammonia detoxification by transforming ammonia to glutamine in tilapia. On the other hand, one of the important pathways for ammonia excretion in fish is by transport of ammonia from plasma to ambient water through the gills. Rhbg or Rh glycoproteins are usually considered as crucial ammonia conduits (Weihrauch et al., 2009). In this study, we found that miR-26a is also expressed primarily in the gills. This suggests miR-26a could potentially influence gill ammonia transport mechanisms.

Earlier reports have shown that oxidative damage commonly occurs after high ammonia exposure in fish (Hegazi et al., 2010; Lisser et al., 2017). Thus, we examined whether miR-26a silencing influences oxidative stress response in tilapia. ROS production was decreased, and the activities of SOD, CAT and GPX increased in the brain of tilapia after the silencing of miR-26a. These data suggest that miR-26a silencing could strengthen the oxidative stress response in tilapia. miR-26a loss of function may play a protective role, helping tilapia combat against ammonia stress.

HSP70 is a member of the important stress-protein families and its expression is remarkably induced in response to various stresses including extreme temperature, UV irradiation, infection, and toxic chemicals (Wu, 1995). The up-regulation of *HSP70* in the adaptation to hyper-ammonia stress in fish has been well studied, e.g. mud eel *Monopterus cuchia* (Hangzo et al., 2017), common carp *Cyprinus carpio L* (Sung et al., 2012) and blunt snout bream *Megalobrama amblycephala* (Sun et al., 2016b). GS is an enzyme that plays a crucial role in the metabolism of nitrogen by catalyzing ammonia to glutamine. High GS activities are commonly found in fish in defense against ammonia toxicity (Ip et al., 2001b). Here, we found that miR-26a knockdown leads to increased *HSP70* and *GS* by targeting their 3′-UTR *in vivo*. Nevertheless, ammonia metabolism and toxicity involves multiple pathways. It is unlikely that ammonia tolerance is regulated by one or two genes. *HSP70* or *GS* production is also controlled by multiple mechanisms.

We revealed that miR-26a knockdown is involved in the remission of physiological disorders upon ammonia stress in tilapia. A link between miR-26a and stress responses to ammonia exposure was confirmed. miR-26a knockdown leads to the up-regulation of the intracellular level of *HSP70*/*GS* by directly targeting their 3′-UTR. Our work increases the available information about the regulation of miR-26a and will help us gain insight into the posttranscriptional regulation mechanism of stress response in fish. However, future studies with defined end-point (e.g. loss of equilibrium or death) are needed to verify the role of miR-26a in ammonia tolerance, and other functional target genes of miR-26a involved ammonia tolerance will also need to be validated.

## MATERIALS AND METHODS

### Animals

Nile tilapias (mean weight=13.18±2.7 g) were captured from the fishery center of Shanghai Ocean University, Shanghai, China. Animals were maintained in a water recirculation system in 100 l tanks at approximately 25°C under a 12 h light: 12 h dark regimen for at least 2 weeks. No attempt was made to separate the sexes. This research was conducted according to the Guide for the Care and Use of Laboratory Animals of China.

### Distribution of miR-26a in the different tissues

Tissue (heart, liver, brain, gill and muscle) of tilapia (*n*=3 fish) that were maintained in freshwater were sampled. All the samples were snap-frozen in liquid nitrogen immediately and kept at −80°C until analysis. Total RNA and miRNA samples were isolated using Trizol reagent (Invitrogen) and the miRNeasy kit (Qiagen) following the manufacturers' recommendations. To measure the miRNA expression, RNA was reverse-transcribed with miRNA-specific stem-loop primers. The expression of miRNA was then analyzed using Taqman Advanced miRNA assays (Applied Biosystems, Foster, USA) with specific miRNA primers and 18S rRNA was used as an internal control. The relative miRNA expression was calculated using the standard 2^–ΔΔCt^ method and each sample was analyzed in triplicate.

### Ammonia exposure and quantification of miR-26a in the brain

High-purity NH_4_Cl (50 g l^−1^) was prepared as a stock solution of ammonia. Ammonia concentrations were maintained by adding a calculated amount of the NH_4_Cl solution. Exposure experiments were conducted in triplicates in 10 l tanks stocked with five fish each. In our previous study, we found that the median LC50 (median lethal concentration) was about 1.5 mg l^−1^ NH_4_Cl at 24 h post exposure, and 1 mg l^−1^ at 96 h post exposure of tilapia (mean weight about 10 g). Tilapia were therefore exposed to different ammonia concentrations (0, 0.5, 1.0, and 1.5 mg l^−1^) for 6 h. Meanwhile, tilapia were exposed to 1.0 mg l^−1^ ammonia concentration for 0, 6, 12, and 24 h, respectively. About 90% of the solution was changed every 12 h. To ensure that the gut was empty, food was withdrawn 48 h prior to the experiments. Brain tissues (*n*=9 fish) were sampled for each condition and miRNA expression was analyzed as mentioned above.

### miR-26a knockdown and physiological analysis

Fish were injected with PBS, nonspecific scrambled antagomir (Scr antagomir) or miR-26a antagomir at a dose of 60 mg kg^−1^ body weight via the tail vein. The group injected with PBS served as control wild type (Wt). For each group, at least 20 fish were injected. The fish were anesthetized with MS-222 (150 mg l^−1^; Sigma-Aldrich) before injection. To check the injection effect on the expression of miR-26a, the level of miR-26a in the brain was measured 8 h or 16 h after injection without ammonia exposure (*n*=6 fish). After 48 h, these injected fish were exposed to 1.0 mg l^−1^ ammonia.

Blood samples were collected to measure ammonia and [Cl^−^]/[K^+^] content, and brain samples were collected to measure glutamine concentration, ROS and antioxidant enzyme activities at 8 h or 16 h after ammonia exposure. Blood or brain that were taken from three fish were mixed as one sample, two samples were collected at each time point. All the analyses were performed in triplicate.

Blood was drawn from the caudal vessels with heparinized syringe and needle. Ammonia content in the blood was determined by an enzymatic kit (R-Biopharm AG, Darmstadt, Germany) based on the glutamic acid dehydrogenase method. [Cl^−^] and [K^+^] concentration in the blood were determined according to our previously described procedures using ultraviolet-visible spectroscopy (Zhao et al., 2016).

Glutamine was measured using a Shimadzu LC-6A amino acid analysis system with a Shim-pack ISC-07/S1504 Li-type column (Shimadzu, Kyoto, Japan). Results are shown as μmol g l^−1^ wet mass for brain tissue.

ROS in the brain was detected using 2, 7-dichlorofluoresceindiacetate (2′-7′-DCFH-DA) from Sigma-Aldrich (Choi et al., 2007)*.* After environmental ammonia exposure, the sample lysates were incubated in 2′-7′-DCFH-DA at 37°C with gentle rocking. Following 3 h of incubation, they were washed with phosphate buffered saline solution. The emission intensity of 2, 7-dichlorofluorescein was detected at 525 nm.

SOD, GPX and CAT activities in brain were determined using the Total Superoxide Dismutase Assay Kit (Beyotime, Shanghai, China), commercial Detection Kit A005 and Kit A007-1 (Jiancheng, Nanjing, China), respectively (Ran et al., 2008). SOD analysis was performed based on the ability of SOD to inhibit the reduction of WST-8 Formazan dye by the xanthine oxidase/xanthine reaction. One unit of SOD enzymatic activity was determined as the amount of sample which is needed to achieve 50% inhibition of the rate of WST-8 Formazan dye reduction. To measure GPX, 85 mmol/l GSH (glutathione), 30 mmol l^−1^ NADPH and GR (glutathione reductase) were mixed in the cuvette. The reaction was then initiated by adding tissue extracts and 15 mmol l^−1^ R-OOH provided by the kit. GPx activities were measured following the absorbance of NADPH at 340 nm. The reaction was as follows:
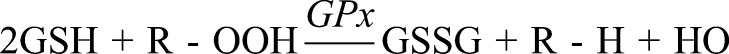

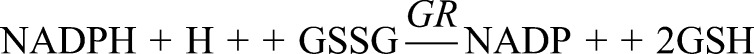


To measure CAT, 50 mmol l^−1^ potassium phosphate buffer (pH 7.0) and 20 mmol l^−1^ H_2_O_2_ were mixed in the cuvette. Tissue extracts were added to start the reaction. Catalase activities were measured following the decay of H_2_O_2_ at 240 nm, and were expressed as μmol of H_2_O_2_ decomposed per second per milligram protein.

### Prediction of miR-26a target genes

Human Embryonic Kidney (HEK) 293T cells were obtained from the American Type Culture Collection and were grown in Dulbecco's modified Eagle's medium containing 10% fetal bovine serum and antibiotic solution at 37°C in a humidified 5% CO_2_ incubator.

For luciferase reporter assays, the segments of the *HSP70* 3′-UTR/*GS* 3′-UTR were subcloned into the pGL3 basic vector (Promega, Madison, USA) downstream of the luciferase reporter gene by standard procedures. Six base pairs in the UTR region were deleted to construct the pGL3- *HSP70*/*GS* mutant. Next, HEK 293T cells were cotransfected with either wild-type or mutant *HSP70*/*GS* 3′-UTR constructs, together with a mimic of miR-26a (miR-26a mimic) or a mimic-negative control (Scr mimic, with no homology to any known tilapia gene) in 12-well plates (2×10^5^ cells/well) using Lipofectamine™ 2000 transfection reagent as instructed by the manufacturer (Invitrogen). Firefly-Renilla luciferase activities were determined using the Dual Luciferase Reporter Assay System (Promega) (Krek et al., 2005).

We performed an antagomir strategy to analyze miRNA knockdown *in vivo* as mentioned above. Fish were maintained in freshwater. The level of miR-26a and *HSP70*/*GS* was measured 12 h or 24 h after injection. Total RNA and miRNA samples of brain (*n*=6 fish, for each condition) were isolated using Trizol reagent (Invitrogen) and the miRNeasy kit (Qiagen) following the manufacturers' recommendations. To measure the mRNA expression, total RNA was reverse-transcribed using SuperScript III (Takara, Dalian, China). mRNAs were amplified using the SYBR Green PCR Master Mix (Takara) with the following primers: HSP70, 5′GCTGATGGCTTCATCACTCA-3′ (forward), 5′CTCCTCCATCCAGGACTTCA-3′(reverse).GS,5′CCTAATTCCTGCTGCCATGT-3′ (forward), 5′CAGGTGTATCGGAGGTTGGT-3′ (reverse). β-actin was used as an internal control. The relative gene expression was calculated using the standard 2^–ΔΔCt^ method.

### Data analysis

The values were presented as the means±s.d., unless stated otherwise. Two-group analysis was performed by Student's *t*-test (normally distributed data) or the Mann–Whitney *U*-test (non-normally distributed data). For multiple comparison groups, a one-way analysis of variance with the Least-Significant difference post hoc test was performed under homogeneity of variance. Analyses were carried out using the SPSS statistical packages (SPSS, Inc.) or Graph-Pad Prism 5.01 software (GraphPad Software Inc.). The level of statistical significance was defined as a *P*-value <0.05.
